# Circulating extracellular vesicular microRNA signatures in early gestation show an association with subsequent clinical features of pre-eclampsia

**DOI:** 10.1038/s41598-024-64057-w

**Published:** 2024-07-22

**Authors:** Shubhamoy Ghosh, Shanthie Thamotharan, Jeanette Fong, Margarida Y. Y. Lei, Carla Janzen, Sherin U. Devaskar

**Affiliations:** 1 Department of Pediatrics, David Geffen School of Medicine, University of California, 10833, Le Conte Avenue, MDCC-22-412, Los Angeles, CA 90095 USA; 2 Department of Obstetrics & Gynecology, David Geffen School of Medicine, University of California, Los Angeles, CA 90095 USA

**Keywords:** Paediatric research, Neonatology

## Abstract

In a prospective cohort of subjects who subsequently developed preeclampsia (PE, n = 14) versus remaining healthy (NORM, n = 12), early gestation circulating extracellular vesicles (EVs) containing a panel of microRNA signatures were characterized and their biological networks of targets deciphered. Multiple microRNAs of which some arose from the placenta (19MC and 14MC) demonstrated changes in association with advancing gestation, while others expressed were pathognomonic of the subsequent development of characteristic clinical features of PE which set in as a late-onset subtype. This panel of miRNAs demonstrated a predictability with an area under the curve of 0.96 using leave-one-out cross-validation training in a logistic regression model with elastic-net regularization and precautions against overfitting. In addition, this panel of miRNAs, some of which were previously detected in either placental tissue or as maternal cell-free non-coding transcripts, lent further validation to our EV studies and the observed association with PE. Further, the identified biological networks of targets of these detected miRNAs revealed biological functions related to vascular remodeling, cellular proliferation, growth, VEGF, EGF and the PIP3/Akt signaling pathways, all mediating key cellular functions. We conclude that we have demonstrated a proof-of-principle by detecting a panel of EV packaged miRNAs in the maternal circulation early in gestation with possibilities of biological function in the placenta and other maternal tissues, along with the probability of predicting the subsequent clinical appearance of PE, particularly the late-onset subtype.

## Introduction

Pre-eclampsia is a systemic disorder that has devastating health consequences for the mother and baby, during and after a pregnancy. More recently, pre-eclampsia has become a major contributor to the exponential increase in maternal mortality world-wide even in well-resourced nations such as the United States. While many investigations have targeted uncovering the etiology and pathophysiology of the disease, there are almost no interventions that have been successful in reversing the condition in time to save the mother and baby from both short-term and long-term consequences. Many attempts have been made to identify non-invasive biomarkers such as unfolding of proteins detected in urine^1,2^, and detection of either a protein or metabolite panel in the urine^3^. More recently, a multi-center clinical trial identified a panel of circulating transcripts in women with pre-eclampsia^4^. In addition, there are several studies examining cell-free non-coding transcripts in maternal circulation towards diagnosing pre-eclampsia in a non-invasive manner^5,6^, including the most recent report^7^.

The pathophysiology of pre-eclampsia is considered to be due to faulty remodeling of the uterine spiral arteries resulting in increasing resistance to maternal blood flow into the placenta, which in turn creates a hypoxic-ischemic microenvironment interrupting adequate nutrient and oxygen delivery to the developing fetus^8^. Depending on the severity, clinical features of pre-eclampsia emerge early or late during pregnancy. The early presentation, after 20 weeks gestation, is the more severe form with a propensity of causing a multi-system (thrombocytopenia, liver disease, vasculitis, kidney involvement, and brain involvement) disorder in the mother along with fetal growth restriction. On the other hand, the late presentation, which is classically detected after 34 weeks gestation, is the more frequently encountered disease state that may or may not involve spiral artery remodeling or multi-systems in the mother nor always produce fetal growth restriction^9,10^. Given the seriousness of this condition, more so the early onset sub-type, early introduction of low dose aspirin has been the mainstay in preventive therapy without a major impact on the outcome of preeclampsia^11^. While many other modalities such as statins have been investigated, no successful intervention has been discovered^12^. This may be due to the lack of well-defined endophenotypes that contribute towards necessary stratification in clinical trials. Thus, currently management is close surveillance and monitoring with early delivery of the baby and placenta, which is thought to ameliorate or at least reduce the symptoms associated with the maternal disorder. This mode of treatment, while aiming to protect maternal health, can lead to the detrimental effects of a premature birth including lung immaturity and neurodevelopmental delays in the baby^13,14^. Given this scenario, it is imperative to continue the search for biomarkers that can reasonably predict the disorder early, so that a window of opportunity can be found in which preventive measures can be investigated with the hope of reversing or at least ameliorating the disorder.

Circulating extracellular vesicles have been shown to play a key role in pregnancy^15–18^ and in complications of pregnancy including preeclampsia^19–22^. These EVs emanating from the placenta package heterogeneous content consisting of bioactive proteins, microRNAs, mRNAs, tRNAs and lipids^15,16^. These circulating EVs provide a vehicle of stable transport for their cargo from the tissue of origin towards communicating biologically with various other destination tissues. Circulating EVs released from the placenta carry a unique cargo of miRNAs, which have a propensity of demonstrating differential expression during different phases of pregnancy^23,24^ and related to certain complications^23,24^, such as gestational diabetes mellitus or preeclampsia^22,25,26^. It is thought that these differentially expressed miRNAs play a role in the disrupted pathophysiology unique to certain pregnancy complications. MicroRNAs are a subclass of small (19–24 nucleotides) non-coding transcripts that complementarily sequence align with 3ʹ-untranslated regions of mRNAs and regulate a network of mRNAs by either cleaving them or suppressing translation^27,28^. Most studies have examined cell-free miRNA signatures rather than that encapsulated within EVs^29–33^. Given the stability invoked for these signatures by the encapsulating EVs, it stands to reason that such miRNA signatures can serve as biomarkers in diagnosing and predicting a pregnancy complication such as preeclampsia, thereby averting some of its associated adverse outcomes^34,35^.

To this end, we hypothesized that extracellular vesicles within the maternal circulation will have a cargo of non-coding transcripts that will have the propensity of heralding pre-eclampsia as early as the first or early second trimester of pregnancy. To test this hypothesis, we undertook a single center clinical trial to describe these microRNAs temporally which are packaged within isolated extracellular vesicles from maternal plasma samples. This study was focused on the preeclampsia (PE) group with a gestation matched normal group of a similar sample size.

## Results

### Clinical attributes of the cohort

This study involved 33 participants, including 7 non-pregnant healthy women as the control group. Among the remaining participants, a sub-group of 12 women had healthy normal pregnancies, while 14 women displayed clinical features of preeclampsia. Following 20 weeks or later after 34 weeks gestation, the preeclamptic group exhibited significant differences in systolic, diastolic, and mean blood pressure measurements (systolic/diastolic > 140/90 mm/Hg). Preeclamptic subjects also demonstrated proteinuria (protein:creatinine ratio 1.36 ± 2.27). Proteinuria measured from 24-h urine collection averaged at 253 mg/day. However, no notable dissimilarity was observed in platelet counts or hemoglobin concentration. Moreover, elevated liver enzyme levels were not detected among the preeclamptic subjects. Interestingly, infants born to mothers with preeclampsia exhibited a lower mean body weight compared to those born from normal pregnancies. A majority of the subjects were diagnosed with preeclampsia towards the end of their pregnancy, beyond 34 weeks gestation, indicating that this cohort predominantly presented a milder form of late onset preeclampsia (12 out of 14). Detailed information regarding maternal demographics, fetal data, and other clinical features can be found in Table 1.

**Table 1:** Clinical characteristics of the patient groups with complicated pregnancies and subjects with Normal pregnancy.

	Normal	Preeclampsia	Significance
Number of samples	12	14	
Ethnicity	Hispanic = 4; White/Asian = 8	Hispanic = 5; White/Asian = 10	NA
Pre-pregnancy or pregnancy BMI	25.80 ± 5.34	26.6 ± 6.37	p = 0.7304
BMI classification	Lean = 58.3%; OBS = 25%; OVWT = 16.6%	Lean = 50%; OBS = 35.71%; OVWT = 14%	NA
BP recorded around 1st trimester (~ 12–15 weeks of pregnancy)			
Blood pressure systolic (mm Hg)	115.83 ± 10.28	121.23 ± 12.71	p = 0.2823
Blood pressure diastolic (mm Hg)	73.33 ± 5.69	77.64 ± 12.23	p = 0.3105
Blood pressure mean	87.5 ± 6.81	92.17 ± 11.78	p = 0.2701
BP recorded at 2nd/3rd trimester (when clinically diagnosed with preeclampsia)			
Blood pressure systolic (mm Hg)	117.91 ± 11.5	146.76 ± 10.17*	*p* = *0.0001*
Blood pressure diastolic (mm Hg)	74.25 ± 8.86	93.58 ± 7.34*	*p* = *0.0001*
Blood pressure mean	88.80 ± 9.34	111.31 ± 6.14*	*p* = *0.0001*
Placenta Wt (g)	491.96 ± 90.44	436.740 ± 90.83	p = 0.2385
Birth Wt classification	%SGA = 0%, AGA = 83.3% LGA = 16.6%	%SGA = 14.28%, AGA = 85.71%, LGA = 0%	NA
Birth Wt (g)	3549.83 ± 482.90	3026.79 ± 658.20*	p = 0.032
Gestational age at delivery (weeks)	38.71 ± 1.2	38.21 ± 2.32	p = 0.3932
Gestational age at PE detection (weeks)	Not applicable	29 weeks 5 days to 40 weeks 6 days (range)	NA
24 h protein (mg)/24 h	Not available	253 ± 205.59 (n = 4 available)	NA
Protein/creatinine	0.46 ± 0.53	1.36 ± 2.27	p = 0.3449
AST (U/L)	19.33 ± 7.65	28 ± 16.76	p = 0.3020
ALT (U/L)	21.83 ± 6.60	23.38 ± 26.21	p = 0.9429
Baby sex (male-to-female ratio)	0.714	1.125	NA
Creatinine (mg/dL)	100.85 ± 84.11	57.25 ± 36.13	p = 0.1351
Platelets × 10^9^/L	196.50 ± 69.54	235.93 ± 65.24	p = 0.1491

Numerical values are presented as mean ± SD. For baby sex male/female ratio was calculated.

OBS = obesity, OVTWT = overweight,  AGA = appropriate for gestational age, SGA = small for gestational age, LGA = large for gestational age, AST  = aspartate aminotransferase, ALT =  alanine aminotransferase, NA = not applicable/available.

* represents significance, with values in italics demonstrating the p values.

### miRNA profile of plasma EVs at different trimesters of preeclampsia versus normal pregnancies

**Figure 1:**
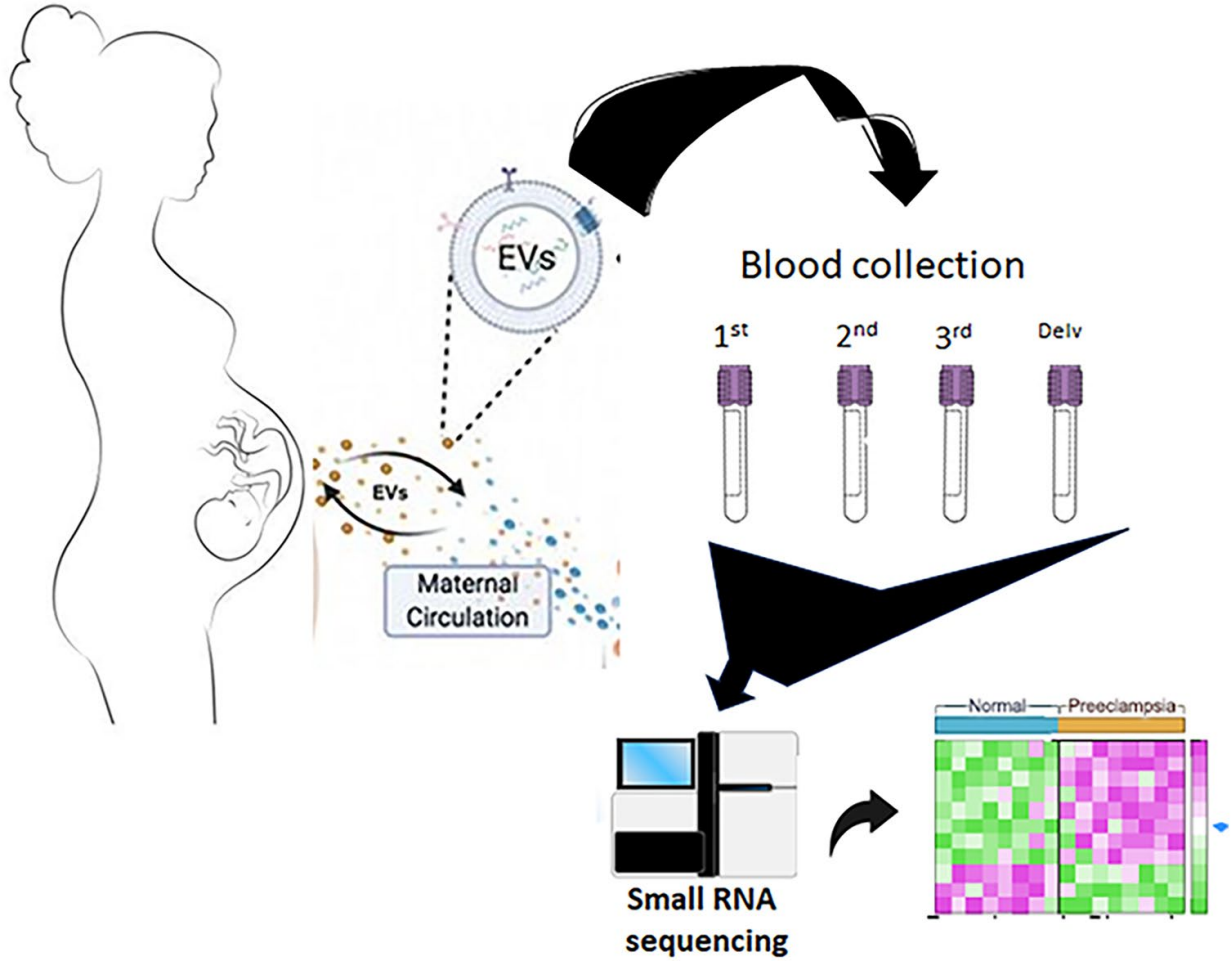
Schematic of the overall experimental workflow.

In our previous study, we conducted a comprehensive investigation of plasma extracellular vesicles (EVs) using immunoblotting, dynamic light scattering (DLS), and transmission electron microscopy (TEM) techniques^23^. Building on this previous work that extensively characterized our EV isolation technique, we currently focused on isolating EVs from peripheral blood samples collected from pregnant subjects with preeclampsia (PE) versus normal pregnancies. Subsequently, we extracted total RNA from these EVs and performed next-generation small RNA sequencing to assess the abundance of expressed miRNAs (Fig. 1).

**Figure 2:**
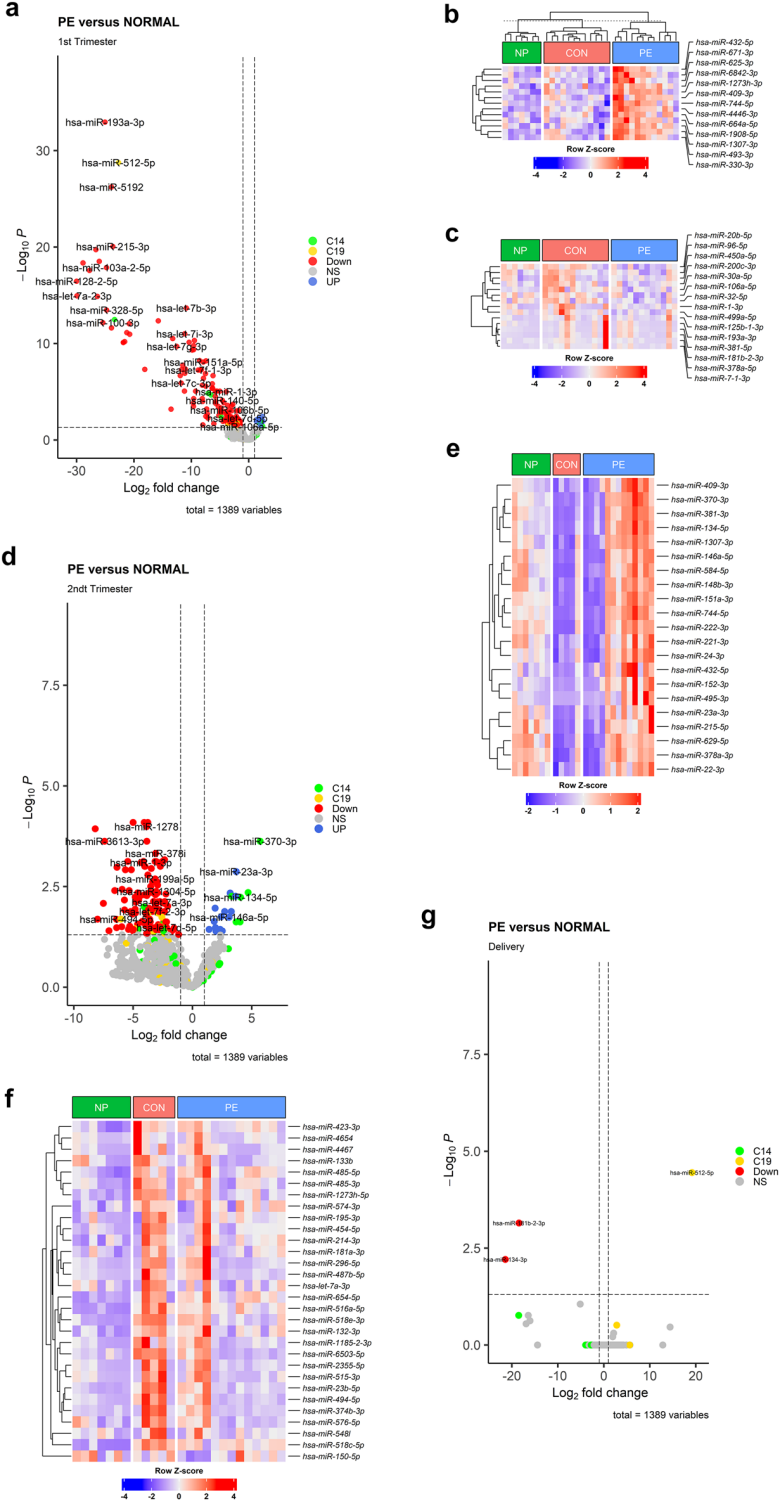
Enhancement of miRNA profile in plasma EVs from preeclamptic subjects when compared to normal pregnancies. Volcano plot demonstrates EV specific differentially abundant miRNAs in preeclampsia (PE) versus normal pregnancies obtained during 1st trimester (**a**), 2nd trimester (**d**), and delivery (**g**), *NS* not significant**.** Heatmap displays abundance of EV derived miRNAs in a blue-red gradient during first (**b,c**) and 2nd (**e,f**) trimesters. All the miRNAs represented had an FDR value ≤ 0.05 and a log2 Fold-Change of ≥ 1 or ≤  − 1. The color bars shown in the heatmap represent the three groups of subjects. Each column refers to an individual subject while each row represents a single miRNA. (**b,e**) demonstrate enhanced miRNAs in preeclamptic subjects versus those with a normal pregnancy (CON) whereas (**c,f**) depict miRNAs that were reduced in PE vs NORMAL (CON). NP = non-pregnant subjects.

During the first to early second trimester of pregnancy, our analysis revealed 148 differentially abundant (DA) miRNAs compared to subjects with normal pregnancies. Among these, 13 miRNAs were enriched in the EVs of the PE group, while 135 miRNAs expressed reduced abundance compared to EVs from women with normal pregnancies (Fig. 2a, Table S1). Specifically, four C14 miRNAs were enriched in PE EVs, while three other C14 miRNAs and four C19 miRNAs displayed reduced abundance (Fig. 2b,c). These findings offer valuable insights into the miRNA profiles within EVs during early pregnancy in preeclamptic subjects, implying potential contributions towards understanding the pathophysiology of the disorder. Additionally, we investigated miRNA abundance in the second and third trimesters, and during delivery (Delv). In the second trimester, we identified 147 DA miRNAs within EVs, with 21 miRNAs enriched in the PE group and 126 miRNAs reduced compared to the gestation-matched normal pregnancy group (Fig. 2d, Tables S2, S3). Moreover, we observed a decrease in several C19 and C14 miRNAs, while six C14 miRNAs were increased in EVs isolated from the PE group (Fig. 2e,f). Notably, there were no significant inter-group differences in miRNA abundance among the third trimester samples (negative data not shown). However, only three miRNAs (has-miR-134-3p, has-miR-181b-2-3p, hsa-miR-512-5p) exhibited a significant increase in abundance from PE subjects at delivery (Fig. 2g, Table S4).

**Figure 3:**
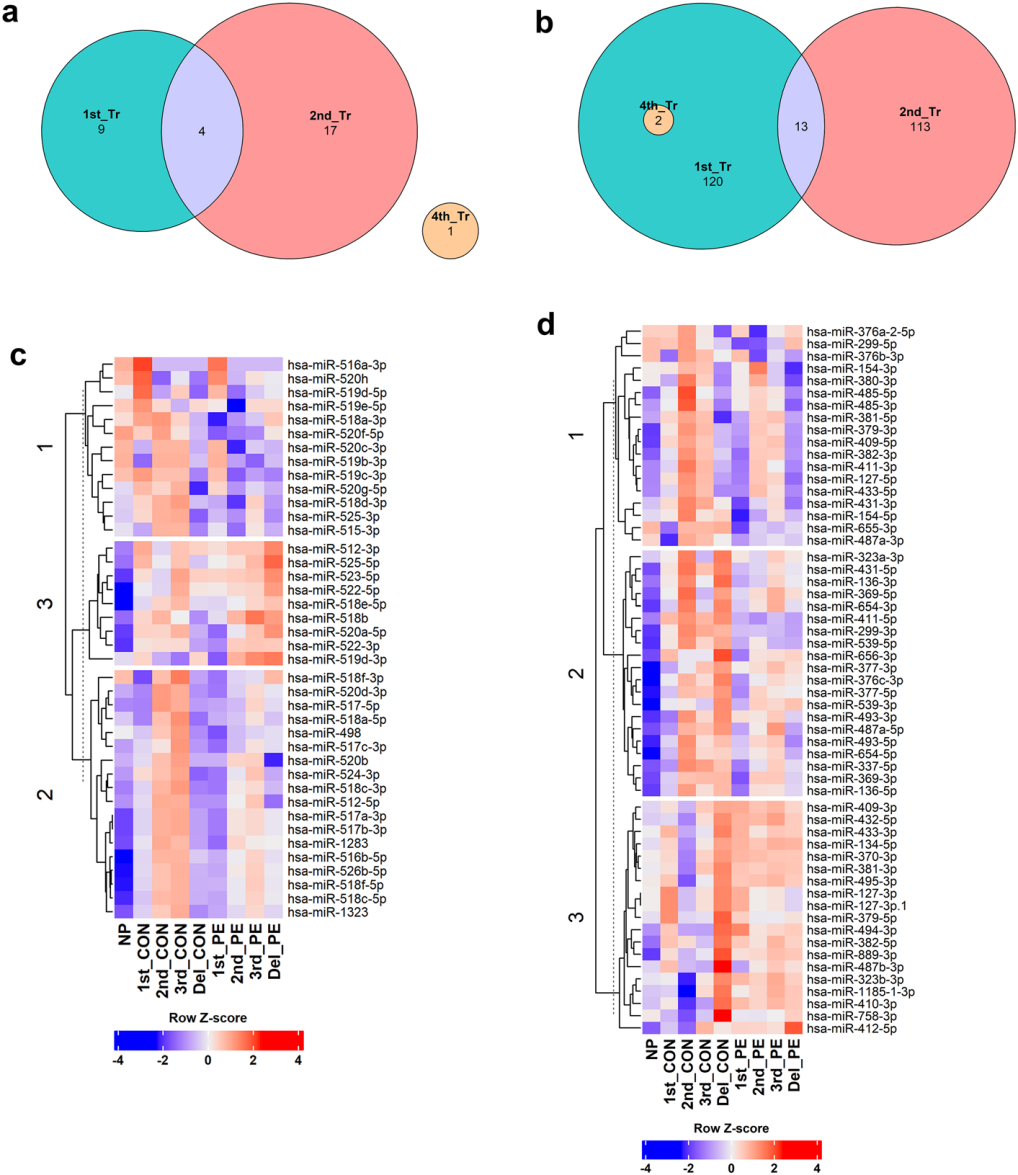
Altered enrichment profile of placenta specific miRNAs during 1st and 2nd trimesters. Venn diagram demonstrating differentially abundant miRNAs during 1st and 2nd trimesters and at delivery. (**a**) Depicts the number of miRNAs enhanced in EVs from preeclampsia while (**b**) shows miRNAs reduced in EVs from preeclampsia relative to normal controls. The 4th sample represents the sample collection at delivery. Heatmap displays abundance of EV derived C19 miRNAs (**c**) and C14 miRNAs (**d**) in a blue-red gradient during different trimesters of pregnancy obtained from NORMAL (CON), PE and non-pregnant (NP) samples. Each row refers to a single miRNA and each column represents each trimester from NP, NORM or PE samples.

Further exploration in PE versus normal pregnancies of EV-specific miRNAs revealed four enriched miRNAs common to both 1st and 2nd trimesters of pregnancy, while nine and 13 miRNAs were exclusively enriched in either the first or second trimesters respectively. Only one miRNA displayed differential abundance at delivery in PE. Concurrently, we observed 13 miRNAs reduced in both the first and second trimesters, while 120 miRNAs were reduced in the first trimester alone and 113 miRNAs were reduced in the second trimester from PE subjects. Two miRNAs were observed to decrease during delivery, which were also noted to be reduced during the first trimester in PE subjects (Fig. 3a,b).

These findings shed light on the dynamic changes in miRNA profiles within EVs observed temporally in preeclamptic subjects and present the possibility of garnering valuable insights into the function of specifically identified and perturbed miRNAs during the progression of PE, ultimately culminating in overt clinical features characteristic of the disease.

### Abundance of placenta specific miRNAs in plasma EVs in preeclampsia versus normal pregnancy

We next conducted a comparison of miRNA abundance packaged in plasma extracellular vesicles (EVs) between preeclamptic and normal pregnancies, with a focus on placenta-specific miRNA clusters, particularly from chromosome 19 (C19) and chromosome 14 (C14) miRNA clusters. Our in-depth analysis unveiled a distinctive temporal pattern for both the plasma EV-derived C19MC and C14MC miRNA abundance in PE subjects compared to normal pregnancies. Specifically, we identified three distinct C19 clusters, with cluster 3 exhibiting higher abundance during the first trimester but was lower in the second and third trimesters in preeclamptic (PE) subjects compared to normal pregnant (NORM) or nonpregnant controls. Conversely, cluster 2 displayed higher abundance of miRNAs compared to non-pregnant control with no significant difference noted in abundance between PE and NORM at any of the trimesters. On the other hand, remarkably cluster 1 demonstrated a lower abundance in PE compared to NORM at all trimesters.

In the case of C14 miRNAs, we have also identified three clusters. Cluster 3 displayed a higher abundance of miRNAs in PE subjects at all trimesters including delivery compared to NORM or non-pregnant subjects, whereas clusters 1 and 2 demonstrated a mixed expression pattern, with reduced abundance of most miRNAs during the second and third trimesters in PE subjects (Fig. 3c,d).

All the placenta specific miRNAs that were differentially abundant (increased or decreased) at all trimesters are listed in Table 2. Expression comparison of all circulating miRNAs during the three trimesters and at delivery is provided in Supplementary Tables (Supplementary Tables S1–S4).

**Table 2:** List of up or downregulated miRNAs at different trimesters of pregnancy and at delivery in PE versus NORM pregnancies.

miRNAs	Up or Dn in PE	Trimester	C14/C19/others	miR target genes	miR target pathways
hsa-miR-512	↓	1st	C19	USF2/PPP3R1 axis	Decorin-induced, preeclampsia
hsa-miR-520a-5p	↓	1st	C19	MMP2/EGFR	EGFR signaling pathway/regulates sFlit-1 secretion (30636550)
hsa-miR-524-3p	↓	1st	C19	NUMB	Notch signaling pathway
hsa-miR-519d-3p*	↓	1st	C19	MMP2	
hsa-miR-379-3p	↓	1st	C14	Rac1/MLK3/JNK/AP-1	Regulate connective tissue growth factors
hsa-miR-381-5p	↓	1st	C14	ROCK2	Cell proliferation, cell migration, cytoskeleton formation
hsa-miR-409-5p	↓	1st	C14		Cell migration and invasion
hsa-miR-409-3p	↑	1st	C14	PPP2CA	
hsa-miR-432-5p	↑	1st	C14	CXCl3/E2F3/AXL	Cell migration and invasion
hsa-miR-493-3p	↑	1st	C14	E2F1	Cell invasion
hsa-let-7a-2-3p	↓	1st	*Let-7*		Proliferation and invasion
hsa-let-7b-3p	↓	1st	*Let-7*		Proliferation and invasion
hsa-let-7c-3p	↓	1st	*Let-7*		Proliferation and invasion
hsa-let-7d-5p	↓	1st	*Let-7*		Proliferation and invasion
hsa-let-7e-3p	↓	1st	*Let-7*		Proliferation and invasion
hsa-let-7f-1-3p	↓	1st	*Let-7*		Proliferation and invasion
hsa-let-7g-3p	↓	1st	*Let-7*		Proliferation and invasion
hsa-let-7i-3p	↓	1st	*Let-7*		Proliferation and invasion
hsa-miR-223	↓	1st	Others	STAT3	Cell survival, cell invasion
has-miR-148a	↓	1st	Others	IGF-IR, IRS1	Apoptosis, cell invasion
has-miR-152	↓	1st	Others	IGF-IR, IRS1	Apoptosis, cell invasion
has-miR-144	↓	1st	Others	PTEN	Proliferation, migration, invasion
has-miR-155	↓	1st	Others	NF-κB	eNOS pathway
hsa-miR-210	↓	1st	Others	HIF-1α, NF-κBp50	Hypoxia
hsa-miR-125a-3p	↓	1st	Others		
hsa-miR-18a-3p	↓	1st	Others	ESR1	Impaired cell invasion
has-miR-370-5p	↓	1st	C14	Endoglin	Motility and proliferation pathway
has-miR-1283	↓	2nd	C19	ATF4	Regulates vascular endothelial dysfunction
Has-miR-515-3p	↓	2nd	C19	MMP3, Vimentin	Invasion
has-miR-516b-5p	↓	2nd	C19		
has-miR-517-5p	↓	2nd	C19	ERK/MMP-2	Alter extra villous trophoblast function
has-miR-518c-3p	↓	2nd	C19	HSD17B1	Inhibit eNOS production causing impaired cell invasion
has-miR-518c-5p	↓	2nd	C19	LRP6	Regulates biological function of trophoblast
has-miR-518f-5p	↓	2nd	C19		
has-miR-520d-3p	↓	2nd	C19	HDAC1	Disrupt vascular endothelium development
has-miR-525-3p	↓	2nd	C19	ARRB1/TXN1	Cell survival
has-miR-134-5p	↑	2nd	C14		
has-miR-370-3p	↑	2nd	C14	Endoglin	Motility and proliferation pathway
has-miR-381-3p	↑	2nd	C14	IGF-1R	Cell growth, apoptosis, migration and invasion
has-miR-409-3p	↑	2nd	C14	PPP2CA	
has-miR-432-5p	↑	2nd	C14	CXCl3/E2F3/AXL	Cell migration and invasion
has-miR-495-3p	↑	2nd	C14	HMGB1	Cell proliferation and migration
has-miR-136-5p	↓	2nd	C14	Rho‑associated coiled‑coil containing protein kinase 1 (ROCK1)	Invasion and migration
has-miR-337-5p	↓	2nd	C14	UBQLN1	Cardiac hypertrophy
has-miR-431-3p	↓	2nd	C14		
has-miR-485-3p	↓	2nd	C14	PGC-1α	Mitochondrial respiration, cell migration and cell invasion
has-miR-485-5p	↓	2nd	C14	PGC-1α	Mitochondrial respiration in cell migration and cell invasion
has-miR-654-5p	↓	2nd	C14	ADAMTS-7	Migration and proliferation
hsa-miR-210	↓	2nd	Others	EFNA3	Cytotrophoblst proliferation/migration
has-miR-512-3p	↑	Delivery	C19	USF2/PPP3R1 axis	Decorin-induced, preeclampsia
has-miR-515-3p*	↑	Delivery	C19	XIAP	
has-miR-498*	↑	Delivery	C19	ZEB2	Inactivates the TGF-β/SMAD and Wnt/β-catenin pathways
has-miR-1283*	↑	Delivery	C19	ATF4	Regulates vascular endothelial dysfunction
Has-miR-1323*	↑	Delivery	C19	TP53INP1	Trophoblast cell viability

Asterisks (*) denote significant differences detected by using p-values rather than FDR values. Third trimester is not displayed as no significant differences emerged.

### Functional enrichment and predictive modeling

**Figure 4:**
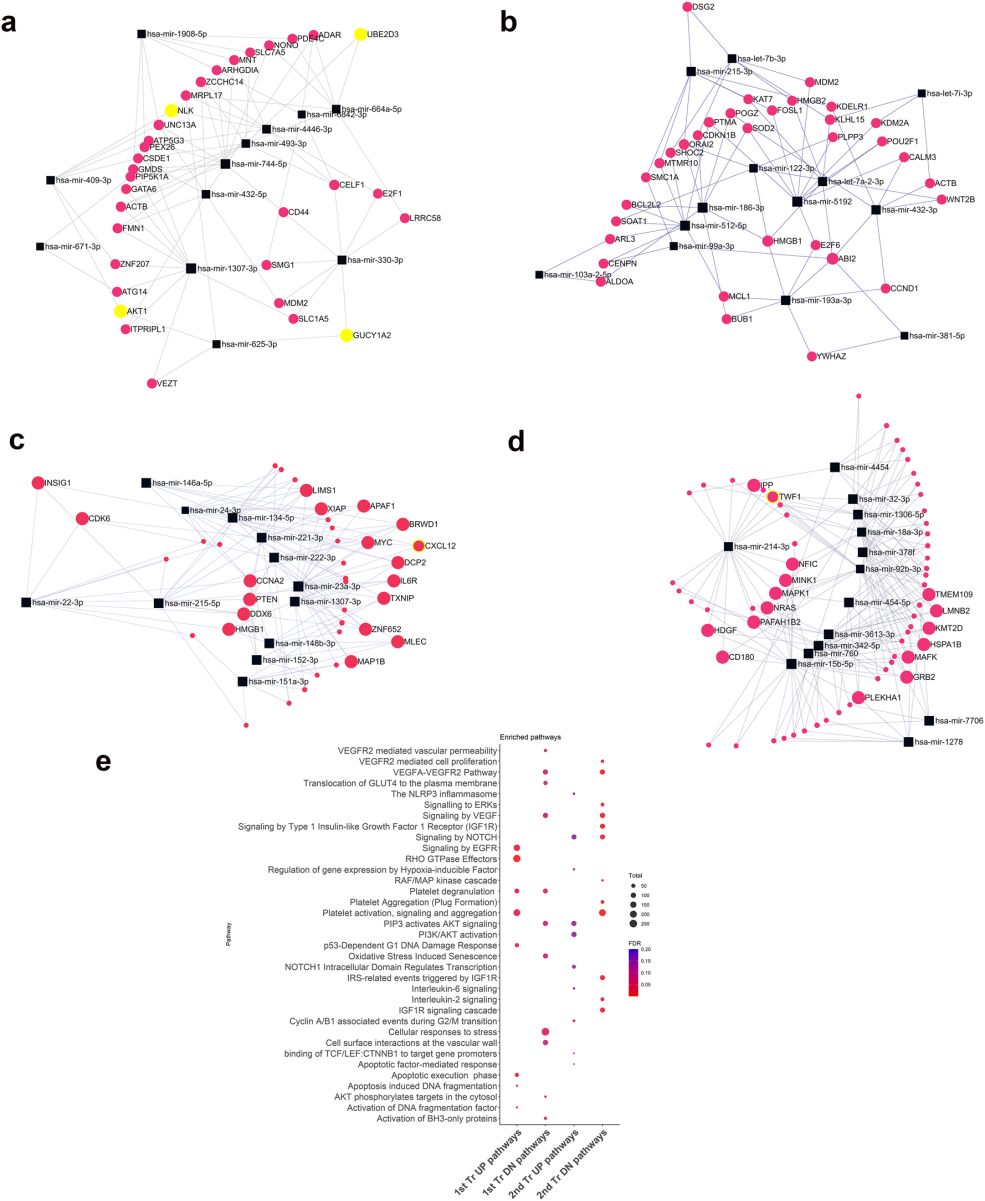
Network analysis of miRNA targets during 1st and 2nd trimesters of pregnancy in preeclamptic subjects: Network plot demonstrating mRNA targets of enriched miRNAs from preeclamptic EVs. First trimester positively (**a**) and negatively enriched (**b**) miRNAs are displayed. Second trimester positively (**c**) and negatively (**d**) enriched miRNAs are also displayed. Dot plot depicts the most significantly enriched functional annotation clusters from the Reactome analysis based on identified targets of EV specific miRNAs (**e**). UP = increased, DN = decreased.

We employed a pathway-centric methodology to investigate the potential influence of currently observed differentially abundant (DA) miRNAs during the early trimesters, aiming to gain further insights into their function. Employing a network-centric approach, we carried out a functional enrichment analysis using TargetScan via the miRNet web tool. Our approach entailed constructing a network that encompassed miRNA-gene interactions, with a focus on the most significantly perturbed DA miRNAs identified within extracellular vesicles (EVs) derived from plasma of subjects who eventually developed preeclampsia (Fig. 4a–d). Our investigation into the predicted miRNA targets revealed a notable enrichment of pathways linked to hypoxia, inflammation, vascular development, as well as regulatory mechanisms governing the cell cycle and cell death (depicted in Fig. 4e) processes.

**Figure 5:**
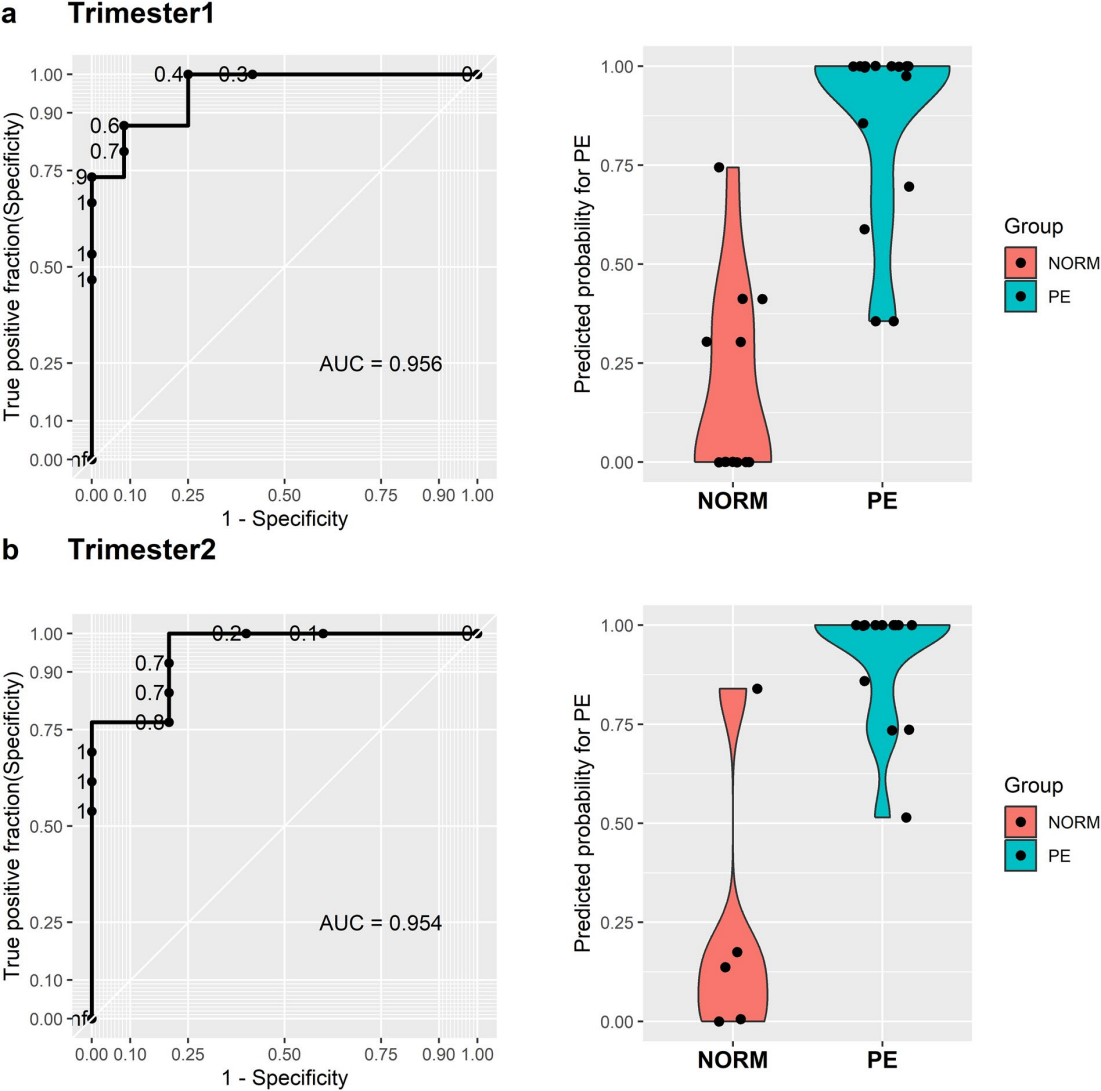
Logistic regression model for prediction of PE in 1st and 2nd trimester: (**a**) First trimester PE (n = 14) versus normal pregnancy (n = 12) receiver operator characteristic (ROC) curve generated using miRNAs isolated from plasma EVs. Violin plot of predicted probability of miRNAs for PE with 0 = low probability and 1 = high probability. (**b**) Second trimester PE (n = 13) versus normal pregnancy (n = 5) miRNA ROC curve and violin plot of predicted probability.

Additionally, we undertook preliminary investigations to establish a connection between miRNA abundance in EVs from the first and second trimesters and the subsequent development of pregnancy complications. We adopted a pooling strategy, considering all detected miRNAs in subjects with preeclampsia (PE) during the first and second trimesters of pregnancy, to create a classifier for early trimester prediction of the subsequent occurrence of clinical PE. Employing elastic-net regularization, we constructed a logistic regression (LR) model trained through leave-one-out cross-validation (LOOCV). By incorporating differentially abundant miRNAs from the first and second trimesters, the model achieved ~ 90% true positive detection rate with an area under the curve (AUC) of 0.956 (as depicted in Fig. 5a,b), signifying the likelihood of developing preeclampsia. The most frequently identified miRNAs in the model for the first trimester included hsa-miR-1307-3p and hsa-miR-520a-5p. In the second trimester, we identified a total of seven predictors which were hsa-miR-1307-3p which was also detected in the first trimester, along with hsa-miR133a-5p, hsamiR-134-5p, hsa-miR-3703p, hsa-miR-381-3p, hsa-miR-494-5p and hsa-miR-495-3p. Among these predictors, hsa-miR-520a-5p is a member of the C19 cluster while hsa-miR-370-3p, hsa-miR-381-3p and hsa-miR-495-3p belong to the C14 cluster.

## Discussion

The focus of our present study was on early detection of a pregnancy associated disorder of preeclampsia. However, during the initial phase of pre-eclampsia, clinical manifestations often remain subtle and perhaps undetected. By the time the disorder advances to its second stage of overt symptoms, detrimental impacts on both maternal health and the developing fetus may have already occurred. This underscores the urgency to investigate new targets as we have done in the present study, that could enable earlier prediction, diagnosis, and may have the propensity to allow stratification of clinical trials with study of endophenotype-targeted novel intervention strategies.

More recently PE has been classified into early onset versus late onset PE sub-types. The etiology of the early onset PE is different from that of the late onset PE, with the former being due to extrinsic factors to the placenta resulting in faulty or incomplete remodeling of spiral arteries during early pregnancy. Factors such as impaired cytotrophoblast invasion, endothelial dysfunction, defective or malformed decidualization and inappropriate immune response to allogeneic fetuses contribute to the early onset PE, thereby contributing to the complexity of the disorder. In contrast the late-onset PE is related more to intrinsic placental factors in response to growth and aging of the placenta thereby restricting intervillous perfusion^36,37^. Whether factors that are integral to the early onset subtype also play a role, albeit to a lesser extent in the late onset subtype is unknown. It is this lack of clarity in the late onset subtype of PE that instigated our current study and its observations. Recent studies have reported that microRNAs are involved in the regulation of trophoblastic cellular proliferation, apoptosis, migration, and invasion, thus interfering with placental development and function^38–40^. Various studies based on microarray, RT-qPCR or some limited sequencing analysis, have detected a list of differentially expressed miRNAs in PE placenta and peripheral blood^41–43^. However, only a limited number of these identified miRNAs were consistently demonstrated across these different studies, perhaps related to subject selection, timing of study and other factors^43^. One of the major sources of miRNAs in peripheral blood are found packaged within extracellular vesicles, which serve as fundamental protective vehicles ensuring cell-to-cell communication^44,45^. Hence, in the present study we focused on the miRNAs within EVs to ensure stability of detected placenta specific biomarkers within maternal circulation.

In our previous study focused on gestational diabetes mellitus (GDM), we determined the intricate role of miRNAs originating from plasma derived extracellular vesicles (EVs)^23^ to potentially serve as early indicators of GDM. Along similar lines we now deciphered the miRNA composition within plasma EVs derived from peripheral blood of late onset PE and normal pregnancy subjects. Our findings reveal noticeable differences in the miRNA content in PE subjects who manifest with a distinct temporal progression. As further validation, we also observed perturbations in certain C19MC miRNAs that originate exclusively from placental trophoblasts, akin to previous reports in peripheral blood of preeclamptic patients^46,47^. Noguer-Dance et al*.* and Bentwich et al*.* have shown the evolutionary adaptation of imprinted C19MC miRNA genes, required for refining signaling pathways important for primate placental development^48–50^. Their research has also illuminated the repercussions of C19MC dysregulation, culminating in dysfunctional trophoblast cells, aberrant placentation, and the subsequent onset of preeclampsia. Prior studies have shown a decrease in C19MCs from placental tissue in pregnancy-related complications, such as gestational hypertension (GE) or preeclampsia^51^. Conversely, specific miRNAs were found to be upregulated in the maternal circulation of individuals with PE^52^, demonstrating inverse changes to that seen in the placenta. Hromadnikova et al.^51^, observed correlation between the decreased placental expression of miR-515-5p, miR-517-5p, miR-518b, miR-518f-5p, miR-519a, miR-519d, miR-520a-5p, miR-520h, miR-524-5p, miR-525 and miR-526a, and the development of PE. They also reported downregulation of miR-519a associated with severe PE. Both miR-519 and miR-520 are known to target matrix metalloprotease 2 (MMP2) and consequently suppress the migration and invasion of trophoblast cells^53^. Our prior observation related to upregulation of a related MMP8 within circulating cell-free transcripts obtained early in gestation from subjects with PE^54^ is in keeping with this concept^54^. Our present study results align with these prior findings from cell-free transcripts^54^, as we observed a reduction in placenta specific C19MCs in EVs of individuals with late onset PE during the first and second trimesters, with an increase noted only during delivery (Table 2). Contrary to these findings in PE subjects, typically, C14MCs exhibit an upregulation during the first trimester, but tend towards downregulation in the later stages of normal pregnancy (p < 0.1).

Numerous other studies have indicated that specific miRNAs may regulate critical processes in the trophoblast. miRNA-223 promotes cell survival and invasion by targeting STAT3^55^ while, the miR-148a/miR-152 axis reduces apoptosis and promotes cell invasion via regulation of HLA-G^56^. miRNA-144 also induces apoptosis, inhibits cell proliferation and is involved in development of PE^57^. Other EV packaged miRNAs namely, miR-210 and miR-155 also contribute to PE by regulating inflammation-related pathways^58^, while miR-210 also inhibits cytotrophoblastic cellular migration. In the present study, we observed a significant reduction of miR-223, miR-148a, miR-152, miR-144 and miR-155 during the first to early second trimester of preeclamptic pregnancy, with notable reductions persisting into mid-2nd trimester. However, miR-210 increased in circulatory EVs during 2nd trimester, with a congruent negative enrichment of PIP3/AKT signaling and VEGF-mediated vascular permeability and cell proliferation during first and second trimesters in PE subjects, both of which could potentially disrupt placental vasculature. We also noticed a reduction of cellular responses to stress and IGFR1 mediated signaling cascades that work through the PI3K/AKT axis and are reduced in preeclampsia. In addition, we observed a positive enrichment of NLRP3 mediated inflammasomes, which are implicated in various pregnancy-related dysfunctional states, including preeclampsia. Furthermore, we observed increased activity of RHO-GTPase effectors and IL-2 signaling. Both these signaling pathways have a positive correlation with hypertension^59,60^. We also saw increased activation of EGFR signaling resulting in elevated secretion of antiangiogenic molecules, which promote the adaptive preservation of vascular integrity. These changes in combination can enhance maternal blood flow to the placenta and contribute towards compensation in response to preeclampsia^61^. This may also be pathognomonic of the late onset PE subtype, where inflammation and vascular adaptation to intrinsic forces may come into play rather than solely a defect in the remodeling of placental vasculature that results in an early gestation placental hypoxic microenvironment.

In our present study EVs serve as a surrogate reflecting early gestation placental health, with C19MC and C14MC miRNAs arising from the placenta. In keeping with a potential for trophoblast and vascular maldevelopment also playing a role in the late onset subtype of PE, a recent magnetic resonance imaging study by our group detected deoxygenation with reduced placental perfusion and volume during early gestation in women who subsequently developed the late onset PE subtype when associated with fetal growth restriction/SGA babies^62^. This suggests that even in the late onset subtype, placental vascularity, perfusion, and oxygenation suffer during early gestation, the degree of which predetermines the birth of SGA babies. In our present study, we noted only ~ 14% SGA babies in our PE group, while our controls delivered ~ 17% large for gestational age infants.

Existing research supports elevated levels of circulatory cell-free miR-210 among preeclamptic patients^63^. miR-210’s regulation by NF-κB transcriptional factor p50 and HIF-1α under hypoxia has been validated^64^. This miRNA has been postulated to inhibit cell migration and vascular remodeling, thereby contributing to defective placentation^65^. In our study, we observed a moderate enrichment of miR-210 in preeclamptic subjects during the second (~ fourfold) and third (~ threefold) trimesters. Furthermore, aligning with previous reports^66^, we have also observed moderate to mild downregulation of miR-17, miR-195, and miR-378a-5p. Specifically miR-195 acts via activin/Nodal signaling, a signaling system that controls the growth and differentiation of trophoblasts. Simultaneously, downregulation of miR-378a-5p and miR-376c has been associated with increased trophoblast cell apoptosis^66,67^.

Existing diagnostic methods of preeclampsia primarily depend on elevated blood pressure and the presence of proteinuria, which signifies compromised kidney function. More recently, the FDA has approved the use of a blood estimation of s-Flt:PIGF ratio in the detection of PE in patients^68^. However, this blood test while effective is only disrupted ~ 2 weeks prior to the emergence of clinical features characteristic of PE^69,70^. Nevertheless, an effective means for a much earlier detection of the disease remains elusive. A recent study focused on a select group of patients who were being triaged for symptoms of PE, demonstrated disruption of a panel of miRs detected by miRNA-sequencing from EVs collected only after 20 weeks of gestation. When this miR panel was combined with an abnormal sFlt:PIGF ratio, a high sensitivity and specificity in detecting the severe form of (early subtype) PE was achieved^7^. In contrast to this study, we were able to detect a panel of perturbed miRNAs much earlier in pregnancy between the 12th and 14th weeks of gestation in addition to those detected between the 19th and 22nd weeks of gestation, that predicted the subsequent development of the late onset subtype of PE. The miRNA panel detected beyond 20 weeks, 0 days to 40 weeks, 6 days gestation reported by Morey et al.^7^, consisted of 522-3p, 4732-5p, 516a-5p, 144-3p, 27b-3p, and let-7b-5p. While we too detected differences between the two groups in some of these miRNAs (Table 2), we additionally noted differences in 1307-3p and 520a-5p (C19MC) in the earlier window (12th to 14th weeks gestation) and 1307-3p, 133a-5p, 134-5p, 3703p (C14MC), 381-3p (C14MC), 494-5p and 495-3p (C14MC) during the 19th and 22nd weeks of gestation. Some of the differences in detected miRNA panels between these two studies may be related to the differences in cohort selection and in the endpoints, namely early onset versus late onset PE subtypes. In addition, despite a larger sample size^7^, the timing of blood sample collection/miRNAs detected were different, along with gestational hypertension without symptoms of PE being included in their control group^7^, unlike our cohort which eliminated gestational hypertension from both groups. Their study showed certain gestational hypertension cases to be closer to controls while other cases of gestational hypertension mimicked PE^7^.

The successful development of a predictive test would significantly contribute to the early identification of the condition along with risk assessment, targeted surveillance, and prompt intervention, prior to the development of any clinical features. We have attempted to build a logistic regression model and trained the model using leave-one-out cross-validation (LOOCV). We found the model to detect around 95% of true positive values at first and 2nd trimesters with the most recurrent predictors being miR-1307-3p and miR-520a-5p. Previously miR-1307-3p has been identified as a potential biomarker for the late onset of preeclampsia^71^. This observation aligns and is further validated by our study in EVs, as most subjects in our study group were diagnosed with preeclampsia during the third trimester. miR-1307-3p has shown its capacity to interact with the methyltransferase protein 8 (METTL8), resulting in the inhibition of KDM3A/3B expression and consequent decrease in methylation of histone H3 at the ninth lysine position. This in turn impairs the endoplasmic reticulum unfolded protein response (UPR)-signaling and initiates preeclampsia^72^. This is highly significant as the more recent easy and cheap urinary based test for detection and diagnosis of PE relies on unfolding of certain key proteins to be pathognomonic of this pregnancy associated disorder^73^. In the case of miR-520a-5p, a negative correlation was reported with the onset of PE^74^. However, during the 2nd trimester, our model predicting a subsequent PE outcome employed not a single miRNA but a panel of miRNAs which were differentially enriched beyond that seen in normal pregnancy. Apart from miR-1307-3p, other predictors i.e. miR-133a-3p has been shown to regulate oxidative stress in cardiomyocytes^75^. Alongside, miR-134-5p, miR-370-3p and miR-495-3p inhibit proliferation, migration and invasion of trophoblasts using differing cellular targets^76–78^. miR-381-3p in turn is responsible for polarization of T lymphocytes while miR-494 inhibits the growth and paracrine function of mesenchymal stem cells (MSC) by decreasing the expression of *CDK6, CCND1* and *VEGF* genes^79^. Thus in the current study, all the perturbed miRNAs we detected in early gestation that were associated with the subsequent development of PE, have biological functions that can be disruptive to placental development, vascular function, trophoblastic cell proliferation and cell cycle, along with the generation of an unfolded protein response.

The strengths of our study consisted of being a prospective study that characterized EV packaged miRNAs temporally throughout pregnancy until delivery. The cohort was not preselected for any high-risk factors nor restricted to a group being triaged for features of PE, making this cohort applicable to any other pregnancy cohort. Our distribution of subjects presenting predominantly with late onset PE allowed us to focus on this entity rather than the more severe early onset PE. Further, we characterized a panel of EV packaged miRNAs whose detected targets were biologically relevant. This panel of miRNAs could be utilized in building a prediction model with high probability of predicting the subsequent development of PE (AUC = 0.956). Our study provides a proof of principle which needs to be further tested separately in the two sub-types of PE found in larger cohorts as part of multi-center trials or a network of PE related RNA sequence information made publicly available. On the other hand, the limitations of our present study are that our cohort predominantly consisted of the late onset PE with only 2 cases of early onset PE. This made it difficult to differentiate between the two subtypes and analyze them separately, although a focus on the late onset subtype was plausible. Secondly, our overall sample size was small given it was a single center prospective study where all subjects who sought prenatal care were enrolled and examined temporally throughout pregnancy rather than pre-selecting subjects who were high risk for PE alone. Given the overall incidence of PE (combining both subtypes) is 5–8%, a prospective study such as the one we conducted here tends to yield a small sample size for the late onset PE alone. The normal pregnancy control sample size was matched to that of the PE group. Further, given that PE has been reported to have a higher incidence in socially disparate populations which is unrelated to race or ethnicity, given our limited PE sample size, such sub-analyses could not be performed.

In conclusion, our study verifies that the composition of miRNAs within circulating EVs throughout pregnancy is influenced by gestation, and pregnancies with adverse outcomes, particularly PE, exhibiting a distinct EV miRNA profile. Our findings while providing a proof-of-principle, underscore the potential of a panel of miRNAs encapsulated in EVs to serve as a noninvasive biomarker for early gestation detection of preeclampsia, prior to the emergence of classical clinical features. Further detection of such a biomarker panel provides the basis for risk stratification and detection of endophenotypes.

## Materials and methods

### Collection of clinical samples

Pregnant women were recruited in the first trimester of pregnancy for a prospective cohort study PARENTs, approved by the UCLA Institutional Review Board (IRB:15-001388) (clinicaltrials.gov: #NCT02786420). All research was performed in accordance with the relevant guidelines/regulations of the National Institutes of Health in accordance with the Declaration of Helsinki. Written informed consents were obtained before 11 weeks of gestation and women were assessed at three subsequent study visits during pregnancy and at delivery. Maternal blood samples were collected from non-pregnant women and temporally from pregnant women in the first to very early second trimester (11–14 weeks), mid- to late second trimester (19–22 weeks), third trimester (36 weeks) and at delivery. We retrospectively divided the subjects into healthy pregnancies (n = 12) or pregnancies diagnosed with preeclampsia (n = 14), following delivery. Blood samples were also collected from non-pregnant women (n = 7) to serve as a baseline for the pregnancy samples. The clinical grouping of preeclampsia versus normal pregnancies was achieved based on established criteria as reported previously^23^. Of note, analysis of some samples in second trimester, third trimester and delivery could not be completed due to lack of adequate sample availability at those time points or due to technical issues related to quality of the constructed miRNA library. Additionally, healthy pregnant women did not have 24-h urine protein collected based on blood pressures found within a normal range as an accepted standard clinical practice.

### Blood collection

Peripheral venous blood was collected into 10 ml EDTA-coated tubes and centrifuged immediately at 2000×*g* for 7 min at 4 °C to separate the plasma fraction from the cells. The plasma was then centrifuged at 16,000×*g* for 10 min at 4 °C to remove any remaining residual cells, aliquoted and stored at − 80 °C until extraction of EVs was undertaken.

### Isolation of EVs from maternal plasma

Extracellular vesicles (EVs) were isolated from peripheral blood of pregnant women using a polymer-based precipitation method (EXOQ5TM-1, System Biosciences, Mountain View CA, USA) as described and extensively validated by us previously^23^. This included immunoblotting for specific EV markers, visualization by transmission electron microscopy, dynamic light scattering for size selection, and flow cytometry for detection of placental origin^23^. Briefly, plasma samples (1 ml) were centrifuged to remove cells and cellular debris, followed by incubation with thrombin and centrifugation at 10,000 rpm. The EV-containing supernatant was then incubated with ExoQuick exosome precipitation solution, centrifuged, and the resulting pellet was resuspended in PBS.

### RNA extraction from EVs

Total RNA was extracted from isolated extracellular vesicles (EVs) using the miRNeasy Mini Kit (Qiagen, Valencia, CA) with minor modifications to the manufacturer’s instructions. The supernatant was applied to a Zymo spin column, and on-column DNaseI digestion was performed. The RNA was washed and eluted with Tris buffer, and its quality assessed by the Agilent Bioanalyzer or Tape Station (Agilent, Santa Clara, CA, USA) prior to library preparation.

### Preparation and sequencing libraries

Total RNA (Approximately 6 µl) extracted from EVs was used for preparing miRNA sequencing libraries. For the miRNA libraries, the NEB Next Multiplex small RNA Library prep kit (NEB E7300S; New England Biolabs, Inc, Ipswich, MA, USA) was utilized following the manufacturer’s instructions. These libraries (10 nmol each) were subsequently sequenced using the HiSeq-2500 platform with single-end 50 bp reads (Illumina Inc.; San Diego, CA, USA).

### Analysis of miRNA seq library

We utilized the miRDeep2 package for aligning sequencing reads to known human miRNAs and quantification. The raw sequencing files were processed using the script mapper.pl, which removed adapter sequences, discarded short reads, and aligned them to the hg38 genome using Bowtie software. The quantification of genome-aligned reads against known human miRNAs from miRBase version 21 was performed using the script quantifier.pl with specific options. Raw read counts were normalized for sequencing depth and RNA composition, and differential expression analysis was conducted using DESeq2 software. We applied a log2-fold-change threshold (Log2FC > 1) and false discovery rate (FDR < 0.05) criteria to determine differentially expressed miRNAs. To perform microRNA-target enrichment analysis, we employed miRNet, a miRNA centric visual analytic tool^80^ to perform miRNA set enrichment analysis. Enriched pathways with biological relevance were selected with a significance threshold of FDR < 0.05. The demultiplexed fastq files and expression matrix are available at GEO (Accession number: GSE247251).

### Predictive model for early biomarker discovery

To develop an early biomarker predictive model, we utilized EV derived miRNAs detected during the first or second trimesters of pregnancy. We constructed a logistic regression (LR) model with elastic-net regularization, employing leave-one-out cross-validation (LOOCV) for training. We selected the model that achieved > 80% true positive detection by considering the corresponding miRNAs.

### Statistical analysis

Statistical analysis was conducted using the DESeq2 package together with other R packages like “ggplot2”, “ggrepel”, “ggpubr”, “pheatmap”, “corrplot”, “pROC”, “logistf”, “caret”, and “PRROC”. Log transformation was applied to the data wherever required. Temporal comparisons between the two groups were performed using the non-parametric Kruskal–Wallis one-way analysis of variance, followed by Dunn’s post-hoc test.

### Supplementary Information

Supplementary Table S1.

Supplementary Table S2.

Supplementary Table S3.

Supplementary Table S4.

## Data Availability

All data generated or analyzed during this study are included in this published article (and its Supplementary Information files).
